# Leveraging large language models to generate multiple-choice questions for epilepsy education

**DOI:** 10.3389/fmed.2026.1978708

**Published:** 2026-10-13

**Authors:** Chao Li, Weifan Wang, Chenchen Li, Xuebin Wang, Xinyan Ma, Weibo Zhao, Yuchen Xie, Weitao Wang, Rubing Lin, Zhaoyin Su, Min Wei

**Affiliations:** 1 Department of Emergency, The Brain Hospital of Guangxi Zhuang Autonomous Region, Liuzhou, China; 2 School of Computer Science and Cyber Engineering, Guangzhou University, Guangzhou, China; 3 School of Stomatology, Lanzhou University, Lanzhou, China; 4 The First School of Clinical Medicine, Lanzhou University, Lanzhou, China; 5 School of Basic Medical Sciences, Lanzhou University, Lanzhou, China; 6 Department of Orthopedics, Jiedong District People’s Hospital, Jieyang, Guangdong, China; 7 Department of Orthopedics, Shenzhen Children’s Hospital, Shenzhen, China

**Keywords:** artificial intelligence, epilepsy, generate model, LLM, MCQ, medical education

## Abstract

**Background:**

Large language models (LLMs) are increasingly used to generate medical assessment items, but their reliability for disease-specific question writing remains uncertain. We benchmarked seven LLMs for epilepsy single-best-answer multiple-choice question generation under a standardized blueprint, focusing on structural compliance, clinical accuracy, expert-rated quality and educational usability.

**Methods:**

Each model completed the same 60 prespecified tasks spanning foundational knowledge, clinical cases, treatment decision-making, and diagnostic evaluation and longitudinal management, yielding 420 questions. Tasks were delivered through an identical single-turn prompt without model-specific optimization, web search or external tools. Objective evaluation assessed structural compliance, keyed-answer accuracy, textual characteristics and response time. Two blinded clinical reviewers independently rated content rigor, clarity, distractor quality, educational usefulness, cognitive alignment and overall usability on five-point scales.

**Results:**

After one standardized corrective prompt, all 420 questions met structural requirements, although first-pass compliance ranged from 83.33% to 100%. Keyed-answer accuracy varied substantially, from 61.67% to 98.33%, with Claude-5 achieving the highest accuracy. GPT-5.6 obtained the highest composite expert score (median, 3.75), followed by Claude-5 (3.67) and Kimi-K3 (3.50). Distractor quality was a common limitation, with median scores of only 2–3 across models. Only 2 of 420 questions (0.48%) were judged directly usable without revision, both generated by GPT-5.6; 119 (28.33%) were usable after minor revision and 299 (71.19%) required substantial revision. Longer outputs were associated with better cognitive alignment but not with content rigor or clarity, while response time was unrelated to overall usability.

**Conclusion:**

Contemporary LLMs can reliably reproduce the structure of epilepsy MCQs, but structural compliance and answer accuracy do not ensure educational readiness. Model strengths were multidimensional, and weak distractor construction remained a major barrier to direct use. LLMs may support expert-supervised item drafting, but independent clinical review and subsequent learner-based psychometric validation remain necessary before deployment in medical education.

## 1 Introduction

Single-best-answer multiple-choice questions (MCQs) are widely used in medical education to assess both factual knowledge and clinical reasoning in a standardized manner (1, 2). However, developing high-quality items is labor-intensive, requiring domain expertise, precise control of stem clarity and cognitive demand, and plausible distractors that preserve a single defensible answer (3). This demand has driven growing interest in LLMs for automated medical item generation, extending their established use in question answering and clinical reasoning to educational content development (4). Recent studies across multiple medical specialties have shown that contemporary LLMs can generate structurally valid and clinically relevant MCQs, in some settings approaching faculty-authored items in clarity, relevance and usability (5). However, persistent errors in answer accuracy, distractor quality, difficulty calibration and cognitive alignment, together with substantial methodological heterogeneity across studies, continue to limit direct comparison and generalization (6–8). Among these methodological differences, prompt design is particularly influential, with structured and specialty-specific prompts improving question quality and alignment between intended and expert-rated difficulty, thereby contributing to between-study variability (9, 10).

Epilepsy provides a particularly demanding disease-specific setting for such evaluation because its educational content spans a broad continuum from factual knowledge to diagnostic synthesis and treatment decision-making (11). Affecting approximately 50 million people worldwide, epilepsy encompasses heterogeneous seizure phenotypes, important diagnostic mimics, evolving classification frameworks, multimodal diagnostic interpretation, individualized antiseizure treatment, emergency management and long-term care across diverse patient populations (11–13). These tasks require integration of seizure semiology, diagnostic findings and therapeutic principles (14), with additional complexity introduced by evolving classification and terminology, including the 2025 revision of seizure classification by the International League Against Epilepsy (15). Epilepsy therefore provides a suitable setting for assessing the clinical accuracy, cognitive alignment and educational utility of LLM-generated MCQs. Despite growing evaluation of LLMs in epilepsy-related question answering, differential diagnosis and seizure localization, evidence regarding their ability to generate epilepsy-specific assessment items under a standardized blueprint remains limited.

We benchmarked seven publicly accessible LLMs using a predefined set of epilepsy MCQ-generation tasks delivered through a common prompt. The benchmark covered foundational knowledge, clinical scenarios, therapeutic decision-making, and follow-up and longitudinal management, and combined automated measures of output validity and answer accuracy with blinded expert assessment of item quality, distractor performance, content rigor, educational value and overall usability. This framework enabled a controlled comparison of model performance in epilepsy-specific assessment generation and provides a basis for integrating LLM-assisted drafting with expert review in medical question-bank development.

## 2 Materials and methods

### 2.1 Study design and models

This comparative benchmarking study evaluated seven publicly accessible LLMs for generating single-best-answer MCQs in epilepsy education. Each model completed the same 60 prespecified tasks using an identical prompt and single-turn generation protocol, yielding 420 MCQs. Model outputs were assessed using objective measures and blinded expert evaluation (Figure 1).

**Figure 1:**
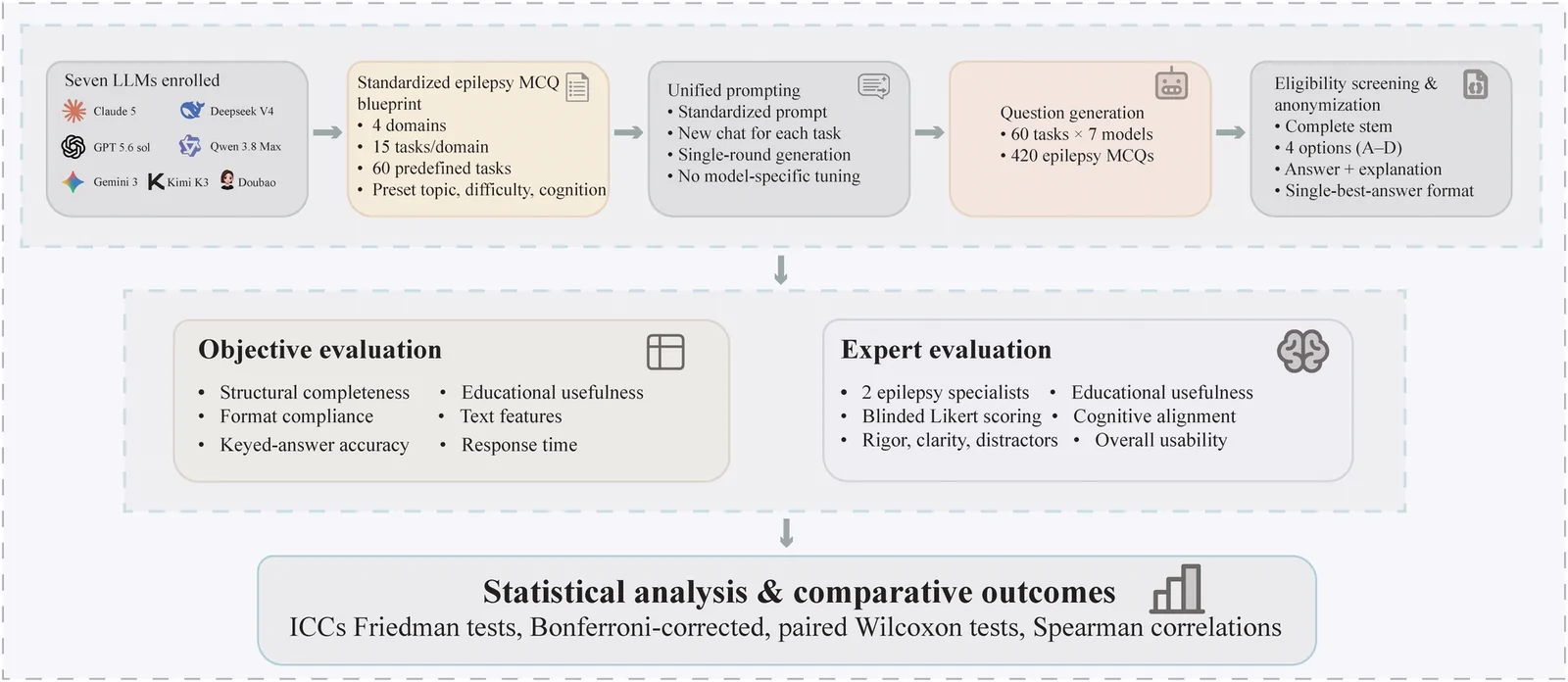
Standardized workflow for epilepsy MCQ generation, multidimensional evaluation and comparative analysis across seven LLMs.

Seven LLMs were evaluated through their official application programming interfaces under standardized generation conditions. The same generation parameters were applied across models wherever technically supported, and no model-specific prompt optimization, iterative revision, web search or external tool use was permitted. Each task was submitted independently in a single-turn interaction, and all raw model outputs were retained for analysis. Model versions, API configurations and detailed generation parameters are provided in Supplementary Material 1.

### 2.2 Epilepsy MCQ blueprint

A prespecified 60-task blueprint standardized content coverage and cognitive demand across models, with 15 tasks assigned to each of four domains: foundational knowledge, clinical cases, treatment decision-making, and diagnostic evaluation and longitudinal management. Each task was defined by a target knowledge point, intended difficulty and cognitive level; full blueprint specifications and task-level settings are provided in Supplementary Material 2.

The blueprint sampled the major domains of epilepsy care, including seizure definitions and classification, epilepsy syndromes and semiology, differential diagnosis, electroencephalographic and neuroimaging interpretation, antiseizure medication management, status epilepticus, recurrence risk and longitudinal care. Topic representation was balanced to minimize overrepresentation of individual content areas. An identical set of 60 task specifications was administered to each model, enabling paired task-level comparisons with model identity as the principal source of variation. The full blueprint is provided in Supplementary Table S1.

### 2.3 Prompting and question generation

A single prompt template was used throughout the study. For each task, the prompt specified the content domain, knowledge point, intended difficulty and cognitive level and required one single-best-answer MCQ comprising a stem, four options (A–D), one correct answer and a concise explanation. Questions were required to contain a uniquely defensible answer and clinically plausible distractors while avoiding ambiguous wording, double negatives, excessive cueing, semantically duplicated options and “all/none of the above” formats. Questions dependent on unavailable images were prohibited; relevant EEG, neuroimaging or laboratory findings were presented textually.

Each task was submitted independently to each model, with one question generated per interaction. If an initial response did not satisfy the required structure, one predefined corrective prompt was permitted. Outputs remaining non-compliant after this attempt were classified as generation failures and excluded from expert quality scoring while remaining in structural analyses. The full prompt templates are provided in Supplementary Methods.

### 2.4 Output processing and reference standard

Questions were included in the analytic set only when they contained a complete stem, four clearly designated response options, a single keyed answer and an accompanying rationale. Items were excluded if essential elements were missing, more than one option could reasonably be defended, or interpretation depended on visual information not provided in the prompt. No post-generation correction or stylistic revision was undertaken. Qualifying items were stripped of model identifiers, combined into a common dataset and presented to expert reviewers in randomized order.

An epilepsy-specific evidence framework was established before outcome assessment to support consistent adjudication of clinical content. Source material comprised current clinical guidelines and consensus recommendations, International League Against Epilepsy classification papers, established neurology and epilepsy textbooks, and selected educational references. For each prespecified task, the framework specified the core concept to be tested, the reasoning required to reach the preferred answer and the boundaries of acceptable clinical interpretation, with newer specialty recommendations taking precedence when sources diverged. A separate reviewer handbook translated these principles into operational rules for objective assessment, anchored expert ratings and resolution of disagreements. Both documents were finalized and locked before the reviewers were exposed to the anonymized model outputs and before formal scoring began.

### 2.5 Objective evaluation

Objective assessment covered output completeness, adherence to the required item format, accuracy of the keyed response, linguistic features and generation efficiency. Completeness reflected whether all prespecified components were present, whereas format adherence captured conformity to the required single-best-answer structure. Answer validity was adjudicated against the predefined evidence framework. A keyed response was classified as incorrect if it conflicted with accepted clinical evidence, was insufficiently supported by the accompanying rationale, or failed to represent a uniquely defensible choice.

Given the dependence of word counts on segmentation methods, textual characteristics were quantified at the character level. Measures comprised stem length, mean option length, rationale length, overall output length and between-option length variability, expressed as the standard deviation across the four response choices. Generation time was calculated from API request submission to receipt of the complete model response and therefore represents end-to-end latency under the study configuration rather than model-intrinsic inference speed.

### 2.6 Expert evaluation

Two clinicians with expertise in epilepsy or clinical neurology and prior experience in medical education independently reviewed all eligible items while blinded to model identity. Before formal assessment, both raters completed a calibration exercise using eight pilot questions excluded from the final analysis to harmonize application of the scoring criteria.

Each item was scored on a five-point Likert scale across six domains: content rigor, clarity, distractor quality, educational usefulness, cognitive alignment and overall usability. Content rigor captured the clinical validity of the stem, response options, keyed answer and rationale; clarity reflected linguistic and logical precision; distractor quality assessed the plausibility of incorrect alternatives; educational usefulness reflected suitability for teaching or formative assessment; cognitive alignment measured consistency with the level specified in the task blueprint; and overall usability indicated the extent to which an item could be deployed in educational practice.

For secondary analyses, overall usability was mapped to three prespecified categories: directly usable (score 5), usable after minor revision (score 4) and requiring substantial revision before use (scores 1–3). These categories were derived directly from the Likert rating and were not scored as a separate endpoint.

Agreement between raters was summarized using intraclass correlation coefficients with 95% confidence intervals. The reliability of the meaning of two ratings was summarized using ICC (2,2). Discordant judgments that affected eligibility, answer validity or usability classification were re-examined against the predefined evidence framework and resolved by consensus. Cases remaining unresolved after discussion were referred to a third senior specialist for adjudication.

### 2.7 Statistical analysis

To assess the potential influence of corrective prompting while preserving the paired design, we repeated the principal quality analyses using only tasks for which all seven models achieved structural compliance on the initial response.

Analyses preserved the paired design created by submitting identical tasks to all seven models. Categorical variables are reported as counts and percentages, with Wilson 95% confidence intervals for key proportions. Continuous variables are presented as mean ± standard deviation or median (interquartile range), as appropriate.

Continuous and ordinal outcomes were compared across models using Friedman tests with task identity as the blocking factor. Significant omnibus tests were followed by paired Wilcoxon signed-rank tests with Bonferroni correction, and Kendall's (W) was reported as the corresponding effect-size measure. Variables showing no between-model variation were summarized descriptively without inferential testing. ICCs were used to assess inter-rater reliability, and Spearman rank correlations examined associations between textual characteristics, response time and expert-rated quality. Correlation analyses were additionally repeated within each model, with P values adjusted for multiple comparisons using the Benjamini–Hochberg false discovery rate procedure. All tests were two-sided, with (*P* < 0.05) considered statistically significant. Statistical analyses were performed using Python (version 3.12.10).

## 3 Results

### 3.1 Generation completion and objective accuracy

The seven LLMs generated 420 epilepsy single-best-answer MCQs across the 60 prespecified tasks. GPT-5.6, Gemini-3.5 and Qwen3.8 met all structural requirements on the initial attempt (60/60 each), whereas first-pass compliance was achieved for 58/60 (96.67%) DeepSeek-V4 outputs, 56/60 (93.33%) Kimi-K3 outputs, 54/60 (90.00%) Doubao-2.1 outputs and 50/60 (83.33%) Claude-5 outputs (Table 1). Thus, 22 of the 420 initial responses required correction. A single standardized remedial prompt resolved every structural deficiency, resulting in complete and format-compliant outputs for all models. No item remained ineligible, and all 420 MCQs proceeded to blinded expert assessment.

**Table 1:** Generation completion, structural compliance and keyed-answer accuracy of epilepsy MCQs generated by seven large language models.

Indicator	GPT-5.6 (*n* = 60)	Gemini-3.5 (*n* = 60)	DeepSeek-V4 (*n* = 60)	Qwen3.8 (*n* = 60)	Doubao-2.1 (*n* = 60)	Claude-5 (*n* = 60)	Kimi-K3 (*n* = 60)
Structurally qualified on first output, *n* (%)	60 (100.00)	60 (100.00)	58 (96.67)	60 (100.00)	54 (90.00)	50 (83.33)	56 (93.33)
Qualified after one standardized remedial prompt, *n* (%)	0 (0.00)	0 (0.00)	2 (3.33)	0 (0.00)	6 (10.00)	10 (16.67)	4 (6.67)
Final structurally complete output, *n* (%)	60 (100.00)	60 (100.00)	60 (100.00)	60 (100.00)	60 (100.00)	60 (100.00)	60 (100.00)
Format compliant, *n* (%)	60 (100.00)	60 (100.00)	60 (100.00)	60 (100.00)	60 (100.00)	60 (100.00)	60 (100.00)
Correct answer accurate, *n* (%) [95% CI]	57 (95.00) [86.30, 98.29]	54 (90.00) [79.85, 95.34]	43 (71.67) [59.23, 81.49]	44 (73.33) [60.99, 82.87]	37 (61.67) [49.02, 72.91]	59 (98.33) [91.14, 99.71]	57 (95.00) [86.30, 98.29]
Included in subjective rating, *n* (%)	60 (100.00)	60 (100.00)	60 (100.00)	60 (100.00)	60 (100.00)	60 (100.00)	60 (100.00)

Structural conformity did not, however, imply equivalent answer accuracy (Table 1). Claude-5 achieved the highest accuracy, with 59 of 60 keyed answers verified as correct (98.33%; 95% CI, 91.14–99.71%). GPT-5.6 and Kimi-K3 each produced 57 correct answers (95.00%; 95% CI, 86.30–98.29%), followed by Gemini-3.5 with 54 (90.00%; 95% CI, 79.85–95.34%). Accuracy was lower for Qwen3.8 (44/60, 73.33%; 95% CI, 60.99–82.87%), DeepSeek-V4 (43/60, 71.67%; 95% CI, 59.23–81.49%) and Doubao-2.1 (37/60, 61.67%; 95% CI, 49.02–72.91%). Notably, Claude-5 combined the lowest first-pass structural compliance with the highest answer accuracy, whereas Qwen3.8 showed perfect initial structural compliance but substantially lower accuracy. These contrasting patterns distinguish adherence to the requested MCQ format from the ability to generate a medically valid answer key.

### 3.2 Model-specific output profiles and response times

Across the 60 matched tasks, all textual characteristics and response times differed among the seven models (Friedman tests, all *P* < 0.001; Table 2 and Figure 2). The clearest differences were observed in explanation length, total response length and generation speed, whereas stem length and option construction varied less substantially.

**Table 2:** Textual characteristics and response times of epilepsy MCQs generated by seven large language models.

Indicator	GPT-5.6	Gemini-3.5	DeepSeek-V4	Qwen3.8	Doubao-2.1	Claude-5	Kimi-K3	Friedman *χ*^2^	Kendall's W	*p*-Values
Stem length	94.22 ± 41.30	114.23 ± 52.25	91.68 ± 43.51	104.27 ± 42.10	96.48 ± 48.07	138.68 ± 64.82	118.17 ± 48.84	103.767	0.288	<0.001
Mean option length	24.75 (13.44, 37.06)	33.50 (23.12, 44.62)	15.38 (6.94, 23.62)	32.25 ± 18.24	29.27 ± 14.70	32.55 ± 15.12	26.25 (14.69, 36.31)	88.191	0.245	<0.001
Explanation length	213.62 ± 51.67	258.87 ± 50.49	191.72 ± 58.76	258.12 ± 60.94	319.50 (277.50, 360.00)	395.42 ± 96.04	302.50 (251.25, 353.50)	212.186	0.589	<0.001
Total response length	409.83 ± 112.22	492.00 (435.00, 565.75)	349.50 ± 93.41	474.00 (394.25, 567.25)	534.00 (413.00, 636.00)	664.32 ± 177.83	517.08 ± 146.55	206.802	0.574	<0.001
Option length SD	4.13 (2.18, 7.86)	3.67 (2.26, 5.24)	3.30 (1.39, 5.37)	3.73 (2.17, 5.81)	3.39 (1.91, 6.31)	6.70 (3.50, 10.07)	5.07 (2.90, 7.85)	28.424	0.079	<0.001
Response time	21.26 (16.75, 28.97)	6.67 (5.33, 7.98)	13.91 (10.62, 22.94)	6.87 ± 1.75	9.84 (7.75, 11.49)	21.65 (17.65, 29.85)	45.58 ± 16.57	289.386	0.804	<0.001

**Figure 2:**
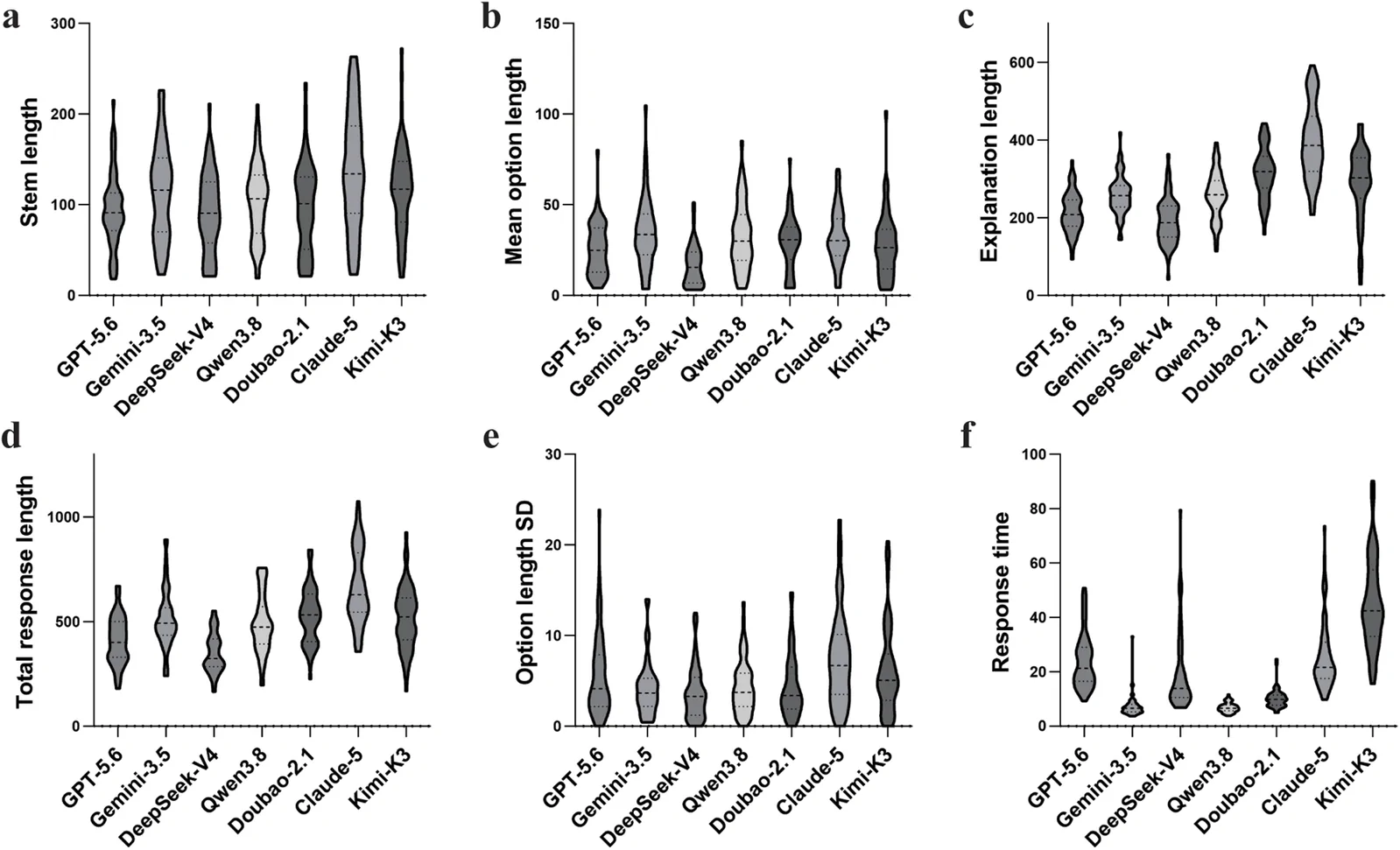
Model-specific textual characteristics and response latency of generated epilepsy MCQs. Distributions are shown across the 60 matched tasks for each model: **(a)** stem length, **(b)** mean option length, **(c)** explanation length, **(d)** total response length, **(e)** option length SD and **(f)** response time. Text length was measured in characters, and latency was measured in seconds from API request submission to receipt of the complete response.

Claude-5 generated the most detailed responses, with average stem, explanation and total response lengths of approximately 139, 395 and 664 characters, respectively. DeepSeek-V4 produced the most concise output, averaging 192 characters per explanation and 350 characters overall. Doubao-2.1 and Kimi-K3 also generated relatively extensive explanations despite producing stems of intermediate length. The distribution of total response length therefore closely mirrored that of explanation length (Figures 2a–d). Options were longest for Gemini-3.5, Claude-5 and Qwen3.8 and shortest for DeepSeek-V4. Differences in option-length balance were comparatively small, although Claude-5 and Kimi-K3 showed greater within-item variation than the other models (Figure 2e).

Generation speed provides the strongest separation between models. Gemini-3.5 and Qwen3.8 completed tasks in approximately 7 s, followed by Doubao-2.1 at approximately 10 s and DeepSeek-V4 at 14 s. GPT-5.6 and Claude-5 each required approximately 21 s, whereas Kimi-K3 required an average of 46 s per task (Figure 2f). Collectively, these results reveal distinct model-specific generation profiles, driven predominantly by the amount of explanatory detail produced and the time required to generate it.

### 3.3 Expert evaluation identifies distractor construction as a common weakness

Expert ratings differed across models in all six quality domains and in the composite score (Friedman tests, all *P* < 0.001; Table 3). The greatest model-level variation was observed for educational usefulness, followed by cognitive-level alignment and the composite score, whereas overall usability showed the least separation.

**Table 3:** Expert-rated quality of epilepsy MCQs generated by seven large language models.

Rating domain	GPT-5.6	Gemini-3.5	DeepSeek-V4	Qwen3.8	Doubao-2.1	Claude-5	Kimi-K3	Friedman *χ*^2^	ICC (95% CI)	Kendall's W	*p*-Values
Content rigor	4.00 (4.00, 5.00)	4.00 (3.00, 4.00)	3.00 (3.00, 4.00)	4.00 (2.75, 4.00)	3.00 (2.00, 4.00)	4.00 (4.00, 4.00)	4.00 (3.75, 4.00)	94.257	0.853 (0.819, 0.881)	0.262	<0.001
Clarity	5.00 (5.00, 5.00)	4.00 (4.00, 5.00)	4.00 (4.00, 5.00)	4.00 (4.00, 5.00)	4.00 (3.00, 5.00)	5.00 (4.00, 5.00)	5.00 (4.00, 5.00)	89.360	0.859 (0.826, 0.886)	0.248	<0.001
Distractor quality	3.00 (2.00, 4.00)	2.00 (2.00, 3.00)	2.00 (1.00, 2.00)	3.00 (2.00, 3.00)	2.00 (2.00, 3.00)	2.00 (2.00, 3.00)	3.00 (2.00, 3.00)	96.194	0.819 (0.778, 0.853)	0.267	<0.001
Educational usefulness	5.00 (4.00, 5.00)	4.00 (3.00, 4.00)	3.00 (2.00, 4.00)	4.00 (4.00, 5.00)	4.00 (3.00, 4.00)	4.00 (3.75, 4.00)	4.00 (4.00, 5.00)	142.770	0.851 (0.816, 0.879)	0.397	<0.001
Cognitive-level alignment	3.00 (3.00, 4.00)	3.00 (2.00, 4.00)	2.00 (2.00, 3.00)	3.00 (2.00, 4.00)	3.00 (3.00, 4.00)	4.00 (3.00, 4.00)	3.00 (2.75, 4.00)	111.022	0.794 (0.748, 0.832)	0.308	<0.001
Overall usability	3.00 (3.00, 4.00)	3.00 (3.00, 4.00)	2.00 (2.00, 3.00)	3.00 (2.75, 4.00)	3.00 (2.00, 3.00)	3.00 (3.00, 4.00)	3.00 (3.00, 3.00)	81.725	0.836 (0.795, 0.868)	0.227	<0.001
Mean score	3.75 (3.50, 4.50)	3.33 (3.00, 4.00)	2.83 (2.50, 3.17)	3.33 (3.00, 3.88)	3.00 (2.62, 3.50)	3.67 (3.29, 4.04)	3.50 (3.29, 3.83)	127.523	0.827 (0.786, 0.860)	0.354	<0.001

GPT-5.6 achieved the highest composite score, with a median of 3.75, followed by Claude-5 at 3.67 and Kimi-K3 at 3.50. Gemini-3.5 and Qwen3.8 each scored 3.33, while Doubao-2.1 and DeepSeek-V4 received lower composite scores of 3.00 and 2.83, respectively (Figures 3a, c). GPT-5.6 showed the strongest overall profile, combining high content rigor with the highest ratings for clarity and educational usefulness. Claude-5 also performed consistently well and received the highest median score for cognitive-level alignment. By contrast, DeepSeek-V4 received the lowest ratings for cognitive alignment and overall usability.

**Figure 3:**
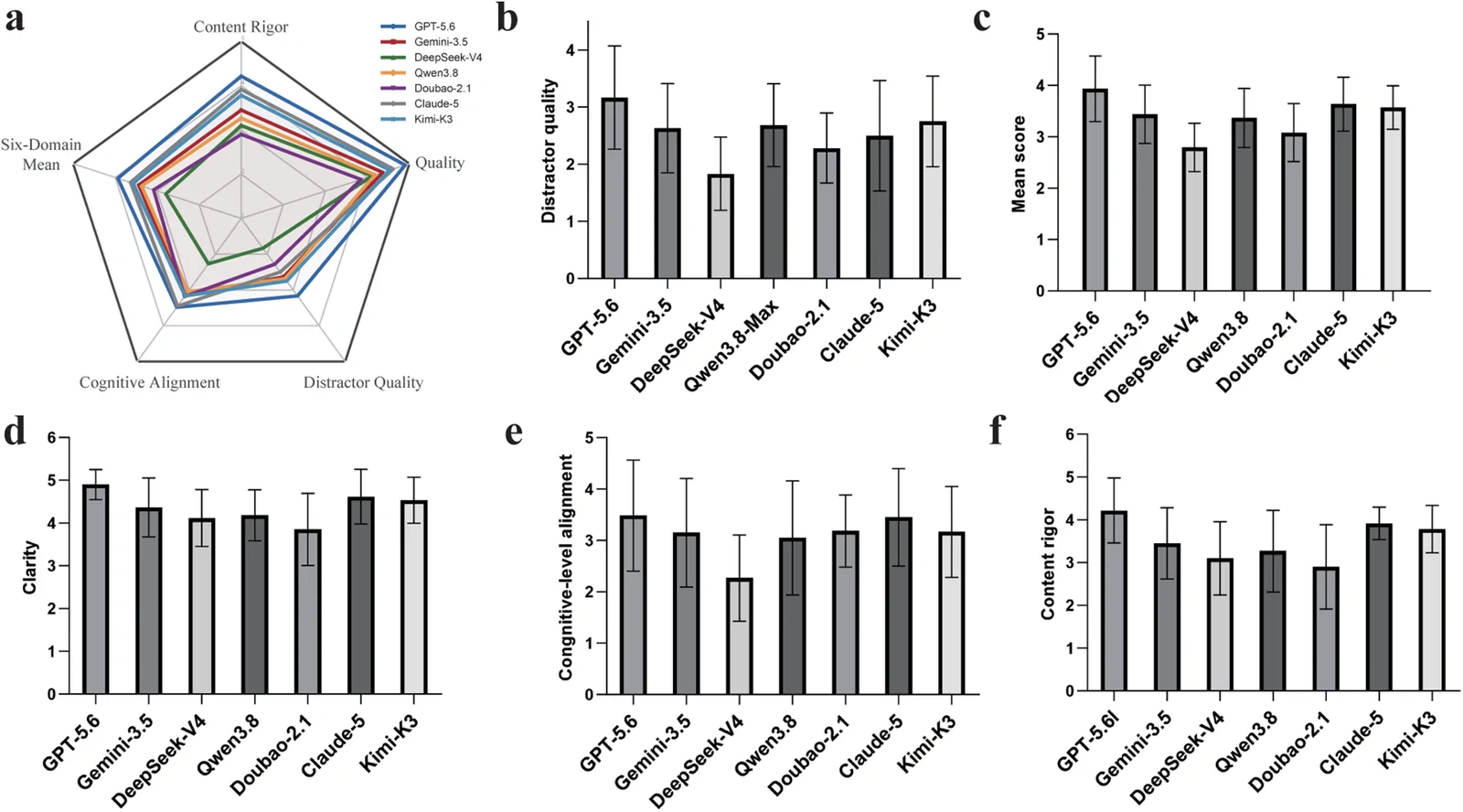
Expert-rated multidimensional quality profiles of epilepsy MCQs generated by seven LLMs. **(a)** Radar plot summarizing model performance across selected expert-rated quality dimensions and the six-domain composite score. **(b–f)** Comparisons of distractor quality, composite mean score, clarity, cognitive-level alignment and content rigor, respectively. Higher scores indicate better expert-rated quality. Each model generated 60 MCQs.

Clarity was generally rated favorably, reaching a median of 5 for GPT-5.6, Claude-5 and Kimi-K3 (Figure 3d). Content-rigor scores were also relatively high for most models, although lower median ratings were observed for DeepSeek-V4 and Doubao-2.1 (Figure 3f). Distractor quality emerged as the principal limitation: median scores ranged from only 2 to 3 across all models, with DeepSeek-V4 showing the weakest distribution (Figure 3b). Overall usability remained modest, with a median of 3 for seven models and 2 for DeepSeek-V4. Thus, although several models generated clear and educationally relevant questions, these strengths did not consistently extend to the construction of plausible distractors or alignment with the intended cognitive level. Twenty-two corrected outputs were distributed across 18 tasks, leaving 42 complete paired tasks for sensitivity analysis. Between-model differences remained significant across all expert-rated dimensions and the composite score (all *P* < 0.001), while the composite-score and overall-usability rankings were unchanged from the primary analysis (Supplementary Table S2).

### 3.4 Few generated items were judged suitable for direct use

As shown in Table 4, overall usability ratings were converted into prespecified expert-derived categories based on Likert-score thresholds. Of the 420 generated MCQs, only 2 (0.48%) were judged directly usable, both of which were produced by GPT-5.6. This corresponded to 3.33% of GPT-5.6 items (95% CI, 0.92–11.36%). None of the items generated by the other six models met the direct-use criterion.

**Table 4:** Expert-derived usability classifications of epilepsy MCQs generated by seven large language models.

Overall usability category	GPT-5.6 (*n* = 60)	Gemini-3.5 (*n* = 60)	DeepSeek-V4 (*n* = 60)	Qwen3.8 (*n* = 60)	Doubao-2.1 (*n* = 60)	Claude-5 (*n* = 60)	Kimi-K3 (*n* = 60)
Directly usable, *n* (%) [95% CI]	2 (3.33) [0.92, 11.36]	0 (0.00) [0.00, 6.02]	0 (0.00) [0.00, 6.02]	0 (0.00) [0.00, 6.02]	0 (0.00) [0.00, 6.02]	0 (0.00) [0.00, 6.02]	0 (0.00) [0.00, 6.02]
Usable after minor revision, *n* (%)	26 (43.33)	20 (33.33)	1 (1.67)	18 (30.00)	13 (21.67)	28 (46.67)	13 (21.67)
Not recommended, *n* (%)	32 (53.33)	40 (66.67)	59 (98.33)	42 (70.00)	47 (78.33)	32 (53.33)	47 (78.33)

Overall, 119 items (28.33%) were considered usable after minor revision, whereas 299 (71.19%) were not recommended without major revision (Table 4). Claude-5 produced the highest proportion of items requiring only minor revision (46.67%), followed by GPT-5.6 (43.33%), Gemini-3.5 (33.33%) and Qwen3.8 (30.00%). The corresponding proportions were lower for Doubao-2.1 and Kimi-K3 (21.67% each) and were particularly low for DeepSeek-V4 (1.67%).

GPT-5.6 and Claude-5 had the lowest proportions of items classified as not recommended, although this category still accounted for 53.33% of the outputs from each model. The proportion increased to 66.67% for Gemini-3.5, 70.00% for Qwen3.8 and 78.33% for both Doubao-2.1 and Kimi-K3. DeepSeek-V4 showed the least favorable distribution, with 59 of 60 items (98.33%) requiring major revision. Thus, despite relative differences among models, most generated items did not meet the threshold for direct educational use. Accordingly, the 0.48% direct-usability estimate reflects attainment of an expert-rating threshold rather than learner-validated educational readiness.

### 3.5 Output length is associated with cognitive alignment rather than content rigor

Exploratory correlation analysis showed that the expert-rated quality dimensions were positively interrelated (Figure 4). Overall usability was most strongly associated with content rigor, educational usefulness and distractor quality, with Spearman coefficients of 0.72, 0.65 and 0.63, respectively; all *P* < 0.001. Because overall usability was included in the six-domain mean score, their strong correlation (*ρ* = 0.88) was considered an expected part–whole association and was not interpreted as an independent empirical finding or evidence of convergent validity. By contrast, clarity was unrelated to cognitive-level alignment, indicating that clearly written questions did not necessarily meet the intended cognitive demand.

**Figure 4:**
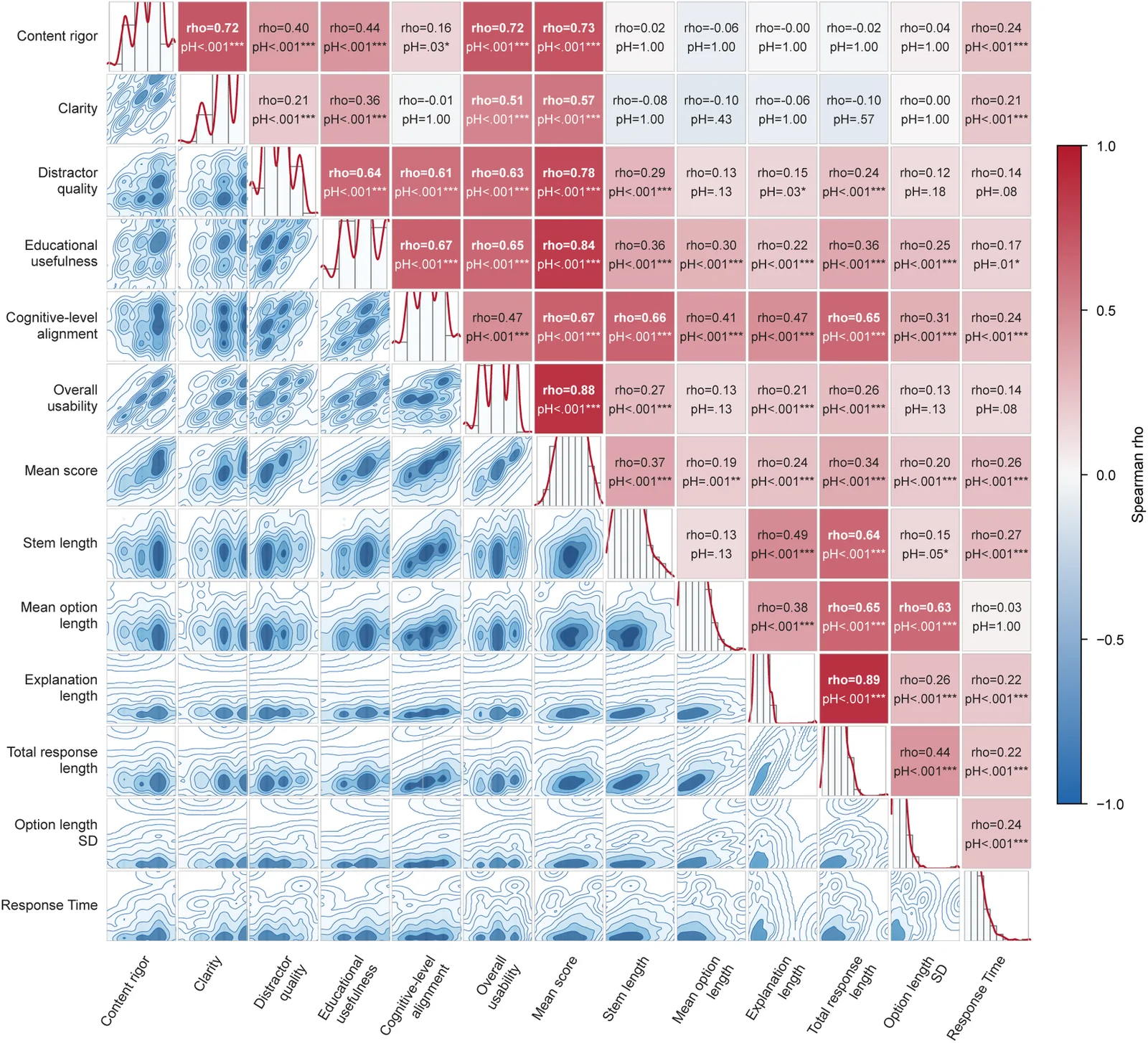
Pairwise associations among expert-rated quality dimensions and generation characteristics. The matrix summarizes pairwise associations among six expert-rated quality dimensions, the overall mean score, textual characteristics, and response time. Diagonal panels show the distribution of each variable, lower-triangular panels display bivariate density contours, and upper-triangular panel's report Spearman's rank correlation coefficients () with corresponding *P* values. Cell colors indicate the direction and magnitude of the correlations, ranging from −1 (blue) to +1 (red). Correlations between the mean score and its component domains represent expected part–whole associations and should not be interpreted as independent evidence of convergent validity.

Text length was associated primarily with cognitive-level alignment. This association was strongest for stem length and total response length, with coefficients of 0.68 and 0.65, respectively; both *P* < 0.001. Explanation length and mean option length showed more moderate associations with cognitive alignment. Longer stems and responses were also associated with greater educational usefulness, but none of the measured length characteristics was related to content rigor or clarity. Total response length was driven mainly by explanation length, with a coefficient of 0.89, followed by mean option length and stem length.

Response time showed weak positive associations with the composite score, content rigor, cognitive-level alignment, clarity and educational usefulness, with coefficients ranging from 0.17 to 0.26. It was not associated with distractor quality or overall usability. Longer response times were also accompanied by modest increases in stem, explanation and total response length. Thus, slower generation tended to produce longer and more cognitively aligned outputs but did not translate into greater overall usability. Model-stratified analyses confirmed that longer stems and responses were consistently associated with better cognitive alignment, whereas associations between response time and most quality outcomes varied across models (Supplementary Table S3).

## 4 Discussion

In this paired benchmark of 420 epilepsy MCQ-generation tasks, all seven LLMs ultimately produced structurally complete items, consistent with evidence that explicit output constraints and standardized blueprints stabilize automatic item generation (5, 10, 16). Structural compliance, however, was decoupled from clinical and educational validity: keyed-answer accuracy ranged from 61.67% to 98.33%, only 2 items (0.48%) were directly usable, and distractor quality remained consistently weak. Claude-5 combined the lowest first-pass compliance with the highest answer accuracy, whereas Qwen3.8 achieved perfect initial compliance but substantially lower accuracy; GPT-5.6, rather than Claude-5, received the highest composite expert rating. Longer and slower outputs were not associated with greater overall usability. Contemporary LLMs therefore reproduce the form of an MCQ more reliably than its substantive requirements, underscoring that instruction following, factual correctness and assessment quality are related but distinct capabilities.

The readiness of LLM-generated MCQs remains context dependent. Studies incorporating specialty source material, structured prompt chaining, fine-tuning or expert pre-deployment screening have reported expert ratings or psychometric properties approaching those of human-written items across several clinical specialties (17–20). A network meta-analysis similarly found no clear differences between GPT-4 and human authors in relevance, clarity or distractor quality, although the certainty of evidence was very low (6). Our markedly lower direct-use rate should therefore be interpreted considering methodological differences: we evaluated unedited, single-turn outputs without model-specific optimization and applied a stringent criterion that reserved direct usability for the highest rating. Consistent with our findings, a prospective high-stakes study showed that ChatGPT-4o reduced item-writing time but generated easier questions with more factual errors and a greater emphasis on lower-order cognition than human authors (7). Conversely, the higher usability reported in a diabetic retinopathy benchmark (16) illustrates that superficially similar scoring frameworks may conceal differences in case mix, evaluation thresholds and rater calibration. Cross-study estimates thus reflect not only model capability but also clinical domain, blueprint design, reference grounding, prompting strategy and human intervention, and should not be regarded as directly comparable measures of deployment readiness.

Model performance was multidimensional rather than strictly hierarchical: GPT-5.6 achieved the highest composite expert score, Claude-5 combined high accuracy with favorable cognitive alignment, and Kimi-K3 produced accurate, clearly written items at substantially greater latency, whereas faster models gained no overall quality advantage. Such heterogeneity is consistent with evidence that model rankings vary across clarity, relevance and distractor quality (16). More importantly, answer accuracy should not be conflated with reliability or trustworthy performance at the level of individual responses. In a recent specialty-examination study, three LLMs achieved overall accuracies of 89.3%–92.9% yet assigned maximum self-reported confidence to 55.6%–87.5% of their incorrect answers (21). This discordance highlights that high aggregate accuracy does not ensure reliable individual outputs, as overconfident errors may still occur and require independent verification. Epilepsy-specific studies further show why accuracy alone is insufficient: models scoring 65–72% on practice board examinations sometimes fabricated reasoning for incorrect answers (22), and GPT-4 distinguished epileptic from functional or dissociative seizures less accurately than experienced neurologists despite improvement with one-shot prompting (23). Prompt engineering has also brought some models close to clinicians in seizure-onset localization, yet expert review identified correct predictions supported by hallucinated knowledge or inaccurate citations, with performance varying by prompt, narrative length and language (24). A correct key may therefore arise from defective reasoning that subsequently contaminates the explanation or distractors. This risk was reflected in our uniformly low distractor scores, a consequential weakness because non-functioning distractors compromise item difficulty and discrimination (25). Constructing plausible but unequivocally inferior alternatives is particularly difficult in epilepsy, where validity depends on classification, temporal sequence, EEG evolution, medication exposure and reproductive context. The revised ILAE framework further increases the risk that superficially plausible options encode outdated terminology (15), complementing broader concerns about unreliable knowledge, bias and limited transparency in epilepsy-focused LLM applications (26). Models may therefore contribute differently to drafting, elaboration or cognitive calibration, but none currently supports unsupervised item generation; every answer, rationale and distractor requires independent expert review against contemporary guidance and its relevant clinical boundary conditions.

Expert ratings further separated linguistic fluency from educational validity. Clarity was unrelated to cognitive alignment, consistent with evidence that grammatically acceptable AI-generated questions may still disproportionately assess lower-order cognition (27). Longer stems and responses were associated with closer cognitive alignment and greater educational usefulness, but not with rigor or clarity. Additional context may support application-level scenarios, yet length cannot substitute for the temporal, semiological or EEG evidence needed to justify an epilepsy item. Response time was likewise only weakly associated with selected dimensions and was unrelated to distractor quality or overall usability. Because latency also captures output length, server load and platform-specific processing, it is better interpreted as an efficiency measure than as evidence of deeper reasoning. These correlations remain exploratory: pooling models with distinct stylistic profiles may confound item-level associations with model-level differences, and multiple comparisons increase the likelihood of chance findings. Future studies should therefore use multilevel models accounting for task and model clustering, prespecify hypotheses and experimentally manipulate stem length, explanation requirements and cognitive instructions to distinguish causal effects from stylistic correlates.

The low direct-use rate also underscores that expert approval is not equivalent to psychometric validation. Learner-based studies have revealed differences in difficulty, discrimination and cognitive demand that are not captured by surface review alone (28, 29). Epilepsy items should therefore be piloted with medical students or neurology residents, with estimation of item difficulty, point-biserial discrimination, distractor functioning, reliability and, where feasible, item-response characteristics. A staged workflow is warranted: LLMs generate candidates from a prespecified blueprint and versioned authoritative sources; automated checks identify structural and key–explanation inconsistencies; epilepsy specialists verify accuracy, terminology, cognitive alignment and every distractor; and surviving items undergo editorial refinement and learner testing. Source grounding, retrieval augmentation and multistep critique may improve performance (28, 30, 31), but cannot be assumed to eliminate error. The present design provides a conservative foundation for this workflow: all seven models completed identical prespecified tasks under a common prompt, outputs were evaluated without model-specific optimization or editing, assessors were blinded to model identity, and a fixed epilepsy-specific framework captured structural, factual, linguistic, efficiency and educational outcomes. These features strengthen paired comparison and reproducibility while preserving the intended evaluation of first-draft performance.

Several limitations constrain interpretation. This benchmark comprised 60 tasks and one response per model–task pair, limiting generalizability and precluding assessment of stochastic variability and test–retest reliability. Accordingly, the observed performance estimates represent a single realization under the specified model/API versions and generation settings reported in Supplementary Material 1 and may vary across repeated generations. Model rankings are also inherently time-sensitive because of rapid version turnover. Subjective evaluation relied on two experts, and usability was defined by a Likert threshold rather than learner performance; without human-authored controls, equivalence or non-inferiority to faculty-written items cannot be inferred. Although the single-turn design strengthened comparability, it may underestimate performance achievable through reference grounding, iterative critique or fine-tuning, while pooled correlations remain susceptible to confounding by model-specific writing styles. Future studies should therefore include repeated generation, multilingual prompting, larger multicenter panels and direct comparisons of single-turn, retrieval-grounded and iterative workflows. Most importantly, generated and expert-written items should be administered prospectively to learners and compared using difficulty, discrimination, distractor functioning and reliability. Domain-specific analyses should examine guideline-sensitive topics—including status epilepticus, pregnancy, medication adjustment and EEG interpretation—and both reference standards and model versions should be explicitly date-stamped to preserve interpretability as epilepsy classifications and LLMs evolve.

## Data Availability

The original contributions presented in the study are included in the article/Supplementary Material, further inquiries can be directed to the corresponding authors.

## References

[1] Schuwirth LW Van Der Vleuten CP. Different written assessment methods: what can be said about their strengths and weaknesses? Med Educ. (2004) 38(9):974–9. 10.1111/j.1365-2929.2004.01916.x 15327679

[2] Parekh P Bahadoor V. The utility of multiple-choice assessment in current medical education: a critical review. Cureus. (2024) 16(5):e59778. 10.7759/cureus.59778 38846235 PMC11154086

[3] Tarrant M Ware J Mohammed AM. An assessment of functioning and non-functioning distractors in multiple-choice questions: a descriptive analysis. BMC Med Educ. (2009) 9:40. 10.1186/1472-6920-9-40 19580681 PMC2713226

[4] Singhal K Azizi S Tu T Mahdavi SS Wei J Chung HW. Large language models encode clinical knowledge. Nature. (2023) 620(7972):172–180. 10.1038/s41586-023-06291-2 37438534 PMC10396962

[5] Artsi Y Sorin V Konen E Glicksberg BS Nadkarni G Klang E. Large language models for generating medical examinations: systematic review. BMC Med Educ. (2024) 24(1):354. 10.1186/s12909-024-05239-y 38553693 PMC10981304

[6] Riehm L Nanji K Lakhani M Pankiv E Hasanee D Pfeifer W. The use of large language models in generating multiple choice questions for health professions education: a systematic review and network meta-analysis. PLoS One. (2026) 21(1):e0340277. 10.1371/journal.pone.0340277 41481658 PMC12758716

[7] Law AKK So J Lui CT Choi YF Cheung KH Kei-ching Hung K. AI versus human-generated multiple-choice questions for medical education: a cohort study in a high-stakes examination. BMC Med Educ. (2025) 25(1):208. 10.1186/s12909-025-06796-6 39923067 PMC11806894

[8] Mufti A Ali K Sharma G Saleem M. Human expertise outperforms artificial intelligence in medical education assessments: MCQ creation highlights the irreplaceable role of teachers. Avicenna J Med. (2025) 15(4):149–156. 10.1055/s-0045-1813040 41473858 PMC12747746

[9] Stadler M Horrer A Fischer MR. Crafting medical MCQs with generative AI: a how-to guide on leveraging ChatGPT. GMS J Med Educ. (2024) 41(2):Doc20. 10.3205/zma001675 38779693 PMC11106576

[10] Kıyak YS Emekli E. ChatGPT prompts for generating multiple-choice questions in medical education and evidence on their validity: a literature review. Postgrad Med J. (2024) 100(1189):858–865. 10.1093/postmj/qgae065 38840505

[11] Blümcke I Arzimanoglou A Beniczky S Wiebe S. Roadmap for a competency-based educational curriculum in epileptology: report of the epilepsy education task force of the international league against epilepsy. Epileptic Disord. (2019) 21(2):129–140. 10.1684/epd.2019.1039 30892268

[12] Manole AM. State of the art and challenges in epilepsy—a narrative review. J Pers Med. (2023) 13(4):623. 10.3390/jpm13040623 37109008 PMC10140944

[13] Singh SP Sankaraneni R Antony AR. Evidence-based guidelines for the management of epilepsy. Neurol India. (2017) 65(Supplement):S6–s11. 10.4103/neuroindia.NI_1027_16 28281490

[14] Boßelmann CM. Seizures, semiology, and syndromes: a narrative review. Seizure. (2021) 92:230–233. 10.1016/j.seizure.2021.09.019 34607271

[15] Beniczky S Trinka E Wirrell E Abdulla F Al Baradie R Alonso Vanegas M. Updated classification of epileptic seizures: position paper of the international league against epilepsy. Epilepsia. (2025) 66(6):1804–1823. 10.1111/epi.18338 40264351 PMC12169392

[16] Qin X Song P Yan Z Qian H Jiang L Wang C. Evaluating large language models for diabetic retinopathy multiple-choice question generation in clinical ophthalmic education. Front Med (Lausanne). (2026) 13:1874243. 10.3389/fmed.2026.1874243 42529502 PMC13416952

[17] Jackson FI Keller NA Kouba I Kouba W Bracero LA Blitz MJ. Large language model clinical vignettes and multiple-choice questions for postgraduate medical education. Acad Med. (2025) 100(10):1163–1166. 10.1097/ACM.0000000000006137 40550116

[18] Gholami S Mummert DB Wilson B Page S Dodhia R Lavista Ferres JM. Leveraging large language models to generate multiple-choice questions for ophthalmology education. JAMA Ophthalmol. (2025) 143(11):955–961. 10.1001/jamaophthalmol.2025.3622 41100119 PMC12532029

[19] Safadi S Amirahmadi R Tlimat A Rovinski R Sun J Lee BW. Quality of human expert vs large language model-generated multiple-choice questions in the field of mechanical ventilation. Chest. (2025) 168(6):1425–1432. 10.1016/j.chest.2025.07.005 40684906 PMC12795417

[20] Hölzing CR Meynhardt C Meybohm P König S Kranke P. Fine-tuned large language models for generating multiple-choice questions in anesthesiology: psychometric comparison with faculty-written items. JMIR Form Res. (2026) 10:e84904. 10.2196/84904 PMC12916093 41707182

[21] Balaban U Erbay B Aktan M Demirkan K. Performance of large language models on the Turkish pharmacy specialty examination: a comparative analysis of accuracy, confidence, and readability. Sci Rep. (2026) 16(1):25822. 10.1038/s41598-026-57001-7 42251118 PMC13482615

[22] Habib S Butt H Goldenholz SR Chang CY Goldenholz DM. Large language model performance on practice epilepsy board examinations. JAMA Neurol. (2024) 81(6):660–661. 10.1001/jamaneurol.2024.0676 38587850 PMC11002770

[23] Ford J Pevy N Grunewald R Howell S Reuber M. Can artificial intelligence diagnose seizures based on patients’ descriptions? A study of GPT-4. Epilepsia. (2025) 66(6):1959–1974. 10.1111/epi.18322 40013839 PMC12169387

[24] Dani M Prakash MJ Rosa F Akata Z Liebe S. Evaluating large language models for diagnostic reasoning from unstructured clinical narratives in epilepsy. Commun Med (Lond). (2026) 6(1):303. 10.1038/s43856-026-01653-z 42174195 PMC13197429

[25] Rezigalla AA Eleragi AMESA Elhussein AB Alfaifi J ALGhamdi MA Al Ameer AY. Item analysis: the impact of distractor efficiency on the difficulty index and discrimination power of multiple-choice items. BMC Med Educ. (2024) 24(1):445. 10.1186/s12909-024-05433-y 38658912 PMC11040895

[26] van Diessen E Van Amerongen RA Zijlmans M Otte WM. Potential merits and flaws of large language models in epilepsy care: a critical review. Epilepsia. (2024) 65(4):873–886. 10.1111/epi.17907 38305763

[27] Nguyen TT Nguyen L Do HTN Nguyen HTT Tong SM. Evaluating cognitive depth of AI-generated multiple-choice questions with bloom’s taxonomy. PLoS One. (2026) 21(2):e0341317. 10.1371/journal.pone.0341317 41758892 PMC12948114

[28] Kaya M Sonmez E Halici A Yildirim H Coskun A. Comparison of AI-generated and clinician-designed multiple-choice questions in emergency medicine exam: a psychometric analysis. BMC Med Educ. (2025) 25(1):949. 10.1186/s12909-025-07528-6 40597998 PMC12210704

[29] Linde P Fichter F Dietlein M Sudbrock F Afshar K Dapper H. Psychometric properties and detectability of GPT-4o-generated multiple-choice questions compared with human-authored items across imaging specialties. NPJ Digit Med. (2026) 9(1):132. 10.1038/s41746-025-02313-7 41507355 PMC12881591

[30] Bedi S Fleming SL Chiang C-C Morse K Kumar A Patel B. QUEST-AI: a system for question generation, verification, and refinement using AI for USMLE-style exams. Pac Symp Biocomput. (2025) 30:54–69. 10.1142/9789819807024_0005 39670361

[31] Thesen T Tuan RL Blumer J Lee MW. LLM-based generation of USMLE-style questions with ASPET/AMSPC knowledge objectives: all RAGs and no riches. Br J Clin Pharmacol. (2026) 92(8):2569–2573. 10.1002/bcp.70119 40483567 PMC13420880

